# Nicotine signals through muscle-type and neuronal nicotinic acetylcholine receptors in both human bronchial epithelial cells and airway fibroblasts

**DOI:** 10.1186/1465-9921-5-27

**Published:** 2004-12-10

**Authors:** Diane L Carlisle, Toni M Hopkins, Autumn Gaither-Davis, Michele J Silhanek, James D Luketich, Neil A Christie, Jill M Siegfried

**Affiliations:** 1 Department of Pharmacology, University of Pittsburgh, Pittsburgh, PA, USA; 2 University of Pittsburgh and Lung and Thoracic Malignancies Program, University of Pittsburgh Cancer Institute, Pittsburgh, PA, USA

## Abstract

**Background:**

Non-neuronal cells, including those derived from lung, are reported to express nicotinic acetylcholine receptors (nAChR). We examined nAChR subunit expression in short-term cultures of human airway cells derived from a series of never smokers, ex-smokers, and active smokers.

**Methods and Results:**

At the mRNA level, human bronchial epithelial (HBE) cells and airway fibroblasts expressed a range of nAChR subunits. In multiple cultures of both cell types, mRNA was detected for subunits that constitute functional muscle-type and neuronal-type pentomeric receptors. Two immortalized cell lines derived from HBE cells also expressed muscle-type and neuronal-type nAChR subunits. Airway fibroblasts expressed mRNA for three muscle-type subunits (α1, δ, and ε) significantly more often than HBE cells. Immunoblotting of HBE cell and airway fibroblast extracts confirmed that mRNA for many nAChR subunits is translated into detectable levels of protein, and evidence of glycosylation of nAChRs was observed. Some minor differences in nAChR expression were found based on smoking status in fibroblasts or HBE cells. Nicotine triggered calcium influx in the immortalized HBE cell line BEAS2B, which was blocked by α-bungarotoxin and to a lesser extent by hexamethonium. Activation of PKC and MAPK p38, but not MAPK p42/44, was observed in BEAS2B cells exposed to nicotine. In contrast, nicotine could activate p42/44 in airway fibroblasts within five minutes of exposure.

**Conclusions:**

These results suggest that muscle-type and neuronal-type nAChRs are functional in airway fibroblasts and HBE cells, that prior tobacco exposure does not appear to be an important variable in nAChR expression, and that distinct signaling pathways are observed in response to nicotine.

## Background

Nicotine, the addictive component of tobacco smoke, signals through its family of receptors, the nicotinic acetylcholine receptors (nAChR). Acetylcholine is the endogenous ligand for these receptors, and has been found in many tissues outside of the nervous system. Non-neuronal nAChR have also been identified in tissues such as the skin, vasculature, and nasal mucosa [1]. nAChR are pentamers that form ion channels permeable to either calcium or sodium. There are several types of nAChR, which are defined by the subunit composition of the receptor. Receptors contain all α-subunits, a combination of α and β subunits, or α, β, ε/γ, and δ subunits [2]. Heteropentamers have been classified as either muscle nAChR, which were first identified at the neuromuscular junction, or as neuronal type, which were discovered in the central nervous system. Homopentamers were also discovered in the CNS and are also considered to be neuronal-type receptors. The adult muscle type receptor contains the α1/β1/ε/δ subunits, with the β1 subunit occurring twice to make the pentamer. The neuronal heteropentamers occur with several different specific subunits, but always with three α and two β subunits, numbered α2 through α6 and β2 through β4. The homopentamer that has been characterized most completely is the α7 pentamer, although recently others have been identified (α8, α9, and α10). The α9 and α10 subunits are unique in that they can form functional homopentamers or can combine together to form a heteropentamer without a β subunit. They are also different from the other subunit combinations examined because nicotine acts as a competitive antagonist to receptors containing the α9 subunit [3].

The ionic permeability of nAChR is dependent upon the subunit composition of the receptor, with some receptors showing preference to either calcium or sodium [4]. However, regardless of initial preference, stimulation of all nAChR by agonist are thought to lead to a calcium influx, either directly through the nAChR channel or due to a change in membrane potential that leads to the opening of calcium L-channels [4]. Extended exposure of nAChR to agonist can lead to receptor inactivation [5]. Again, the degree and severity of inactivation depends upon the subunit composition of the receptor [5].

The primary route of exposure to nicotine is through inhalation, either by active smokers or non-smokers exposed to environmental tobacco smoke. Through inhalation, the lung, in particular, would be exposed to pharmacological doses of nicotine. In addition, receptor inactivation is likely to occur in sensitive receptors, due to the extended length of time that smokers use tobacco [2,5]. This could lead to changes in receptor expression over time; in the brain, it has been noted that the type of nAChR expressed is different in smokers than in never smokers.

Using radiolabeled agonist, we have shown that saturable nicotine binding sites exist in the lung [6]. Other previous studies of the airways exposed to nicotine have shown changes in expression of collagen [7]. *In vitro *data has indicated that airway epithelial cells release GM-CSF upon exposure to nicotine, and activate Akt, a signaling molecular important in cell survival [8,9]. However, although a number of different nAChR subunits are reportedly expressed at the mRNA in human airway cells, [9-12] airway tissue has not been examined for the presence of neuronal-type nAChR at the protein level or for the muscle-type nAChR at the mRNA or protein levels. It is also not known if particular nAChR are more likely to signal through particular downstream pathways when more than one receptor type is present, if the nAChR present in the airway changes after long-term exposure to nicotine, or if calcium influx is responsible for downstream signaling.

In this study, a series of 37 short-term human bronchial epithelial cultures, 25 airway fibroblast cultures, and 2 immortalized bronchial epithelial cell lines were examined by RT-PCR for nAChR expression. We also examined protein expression by immunoblot to determine which subunits are most highly expressed and to determine if appropriate combinations are present at the protein level to form functional receptors. We determined that the nAChR present are functional by examining calcium influx after agonist exposure, and blockade by antagonists. We also show that exposure of airway cells to nicotine leads to activation of downstream signaling pathways. Finally, we examined the nAChR present in HBE and airway fibroblasts derived from smokers, ex-smokers, and never smokers to determine if alterations in nAChRs based on tobacco exposure can be detected.

## Methods

### Primary Airway Cell Culture

HBE cells are cultured from airway biopsies using standard methodology in serum-free medium [13]. Briefly, biopsies are taken from the carina in an area that is normal by appearance by white light bronchoscopy and confirmed using LIFE bronchoscopy. Biopsies are teased apart with forceps and HBE cells are cultured in BEGM (Cambrex Biosciences, Walkersville, MD) for a maximum of two passages on collagen IV coated flasks. This medium is selective for bronchial epithelial cells and at the first passage, cultures are examined and contain 95% or greater epithelial cells. Human airway fibroblasts are derived from bronchial tissue that is minced with scalpels and cultured in DMEM with 10% bovine serum. HBE cells cannot be propagated in this medium. Biopsies were obtained during surgical thoracic resection or bronchoscopy procedures from normal areas of the upper airway. All tissue donors gave informed consent under an approved IRB protocol and answered questionnaires regarding tobacco exposure.

### Airway Cell lines

BEAS2B were all purchased from ATCC and cultured according to ATCC instructions. IB3-1 cells were derived from a cystic fibrosis patient and cultured in Hams medium with 10% serum [14].

### Supplies

All chemicals used were from Sigma (St. Louis, MO) and all supplies were from either Fisher Scientific (Pittsburgh, PA) or PGC Scientific (Frederick, MD), unless otherwise indicated.

### RNA and Protein Isolation

RNA is isolated from cultures using standard guanidinium thiocyanate method [15]. RNA is quantitated using the absorbance at 260 nM and purity is determined using the A260/A280 ratio. After RNA is isolated from the aqueous phase of the solution, protein is extracted from the organic phase, using the method recommended by Invitrogen for the isolation of protein from Trizol. The protein pellet is resuspended in 1% SDS with protease and phosphatase inhibitors added and stored at -80°C. Alternatively, if RNA was not taken from the sample, protein was analyzed from whole cell lysates. Airway cells were scraped into RIPA buffer (150 mM NaCl, 50 mM Tris, pH 7.4, 5 mM EDTA, 1% igepal, 0.5% SDS, 1% deoxycholate) with protease inhibitors 10 μg/ml PMSF, 30 μl/ml aprotinin, and phosphatase inhibitor 1 mM sodium orthovanadate. Lysates were store at -80°C. All protein was quantitated using the BCA assay (Pierce, Rockford IL).

### RT-PCR of nAChR

Primers were developed to unique regions of each subunit and were tested on human muscle and brain RNA purchased from Clontech (Palo Alto, CA). Optimized protocols were then used on RNA from airway cells. All primers span introns and do not amplify DNA. GAPDH or actin is always used as a positive control for RNA integrity. Oligo dT_12–18 _(Invitrogen) was annealed to 1 μg total RNA and reverse transcribed with Superscript II (Invitrogen). The reaction contained 1 μg RNA, 500 ng Oligo dT_12–18_, 50 mM Tris-HCl, pH 8.3, 75 mM KCl, 3 mM MgCl2, 10 mM DTT, 1 mM each dNTP, 200 U superscript. Briefly, total RNA was incubated with oligo dT_12–18 _at 70°C for 10 min. The cDNA produced was then used as a template for PCR using specific primers. Table 1 indicates the primer used and optimized conditions for each subunit. PCR amplification was performed in a 20 μl reaction containing 2 μl of the RT reaction, Taq DNA polymerase (Perkin Elmer), 1X PCR buffer, 1.5 mM MgCl2, 1 mM each dNTP and 1 μM primer. PCR was carried out in a Perkin-Elmer 9700 Thermocycler with 2 min, 95°C denaturation, followed by 30 cycles of 94°C for 30 s, 55°-62°C (see table 1) for 30 s and 72°C for 30 s. Final extension was at 72°C for 5 min. 10 μl of each reaction was run on a 1% TBE gel for analysis. β2 and δ subunits were detected using nested PCR. Primary PCR reactions were carried out as described above. 2 μl of the primary reaction was used as the template for the secondary PCR reaction/second round PCR. Thirty rounds of PCR were carried out at the temperatures listed in Table 1.

**Table 1:** RT-PCR Primers and Conditions

Primer	Optimal Annealing Temperature	Product Size	Sequence
alpha 1*	55	580/505	CGT TCT GGT GGC AAA GCT
			CCG CTC TCC ATG AAG TT
alpha 2*	55	466	CCG GTG GCT TCT GAT GA
			CAG ATC ATT CCA GCT AGG
alpha 3	58	464	CTG GTG AAG GTG GAT GAA GT
			CTC GCA GCA GTT GTA CTT GA
alpha 4	58	444	GGA TGA GAA GAA CCA GAT GA
			CTC GTA CTT CCT GGT GTT GT
alpha 5*	55	525	GAT AAT GCA GAT GGA CGT
			TGA TGG TAT GAT CTC TTC
alpha 6	58	372	GTG GCC TCT GGA CAA GAC AA
			CCT GCA GTT CCA AAT ACA CA
alpha 7	58	375	GGA GCT GGT CAA GAA CTA CA
			CAG CGT ACA TCG ATG TAG CA
beta 1	58	479	CTA CGA CAG CTC GGA GGT CA
			GCA GGT TGA GAA CCA CGA CA
beta 2	62	453	CAA TGG CTC TGA GCT GGT GA
			CCA CTA GGT GTG AAG TCG TCC A
		420	GGC TCT GAG CTG GTG ACA GTA
			CAC CTC ACT CTT CAG CAC CA
beta 3	62	439	TGGAGA GTA CCT GCT GTT CA
			CGA GCC TGT TAC TGA CAC TA
beta 4	58	524	GTG AAT GAG CGA GAG CAG AT
			GGG ATG ATG AGG TTG ATG GT
delta	58	471	CAG ATC TCC TAC TCC TGC AA
			CCA CTG ATG TCT TCT CAC CA
		426	CAA CGT GCT TGT CTA CCA CTA C
			GGT AGG TAG AAG ACC AGG TTG A
gamma		546	CGC CTG CTC TAT CTC AGT CA
			GGA GAC ATT GAG CAC AAC CA
epsilon	55	432	GTA ACC CTG ACG AAT CTC AT
			GTC GAT GTC GAT CTT GTT GA

* From Maus *et al. *[12]

Unique primers to each nAChR subunit were used in PCR reactions. All results were sequenced and compared to the known subunit sequences to confirm that the correct subunit was being amplified.

### Immunoblotting of nAChR

50 μg protein with loading buffer was denatured using 50 mM DTT and heated at 80°C for 15 min. Protein was loaded onto 10% Bis-Tris gels (Invitrogen). Brain lysate (US Biologicals) and lysates from myotubes cultures (gift, Z.Z. Wang, U. Pittsburgh) were used for positive control. Protein was transferred to PVDF (Biorad, Hercules, CA) or Multiblots (ISC Bioexpress, Kaysville, UT). PVDF membranes were blocked for 1 hr with 5% blocker (Biorad) in TBS-T (2.7 mM KCl, 138 mM NaCl, 20 mM Trizma, pH 7.4, 0.1% Tween-20 (Biorad)). Primary antibody was diluted in carrier protein, 5% blocker for PVDF or 0.5% casein (Pierce) for Multiblots and incubated at 4°C overnight, see Table 2 for details. After 4 washes with TBS-T, blots were incubated with appropriate HRP-linked secondary antibody (Santa Cruz, Santa Cruz, CA) at the dilution recommended by the manufacturer for 1 hr in carrier protein as previously. Following 4 more washes with TBS-T, ECL was then done (ECL kit, Amersham Biosciences).

**Table 2:** nAChR Antibodies and Conditions

**Subunit**	**Company**	**Dilution**
alpha-1	Sigma	1:30,000
alpha-2	Santa Cruz	1:1000
alpha-3	Santa Cruz	1:2000
alpha-4	Santa Cruz	1:1000
alpha-5	Sigma	1:15,000
alpha-7	Sigma	1:30,000
beta-1	Sigma	1:10,000
beta-2	Santa Cruz	1:1,000
delta	Wang et al. [30]	1:4000

Antibodies specific to particular nAChR subunits were purchased from either Santa Cruz or Sigma. Antibody specific to the δ subunit was a gift from Dr. Zhu-Zhong Wang, University of Pittsburgh.

### Calcium influx assay

Airway cells were grown in 24-well dishes, with 10,000 cells per well. After 24 hours, medium was replaced with serum-free, prewarmed medium spiked with calcium-45, with a final specific activity of approximately 60 μCi/mM calcium. Drug was added to the wells as indicated, in triplicate. If nAChR antagonists or channel blockers were used, they were added 20 min before the addition of agonist. The calcium ionophore A23187 (Molecular Probes, Eugene OR) was used as a positive control for calcium influx. After incubation at 37°C, plates were put on ice and washed 3 times with ice-cold PBS. Lysis buffer (1% SDS, 0.3 N NaOH) was added. Lysates were transferred to scintillation vials, scintillation fluid added, and counted. Results are presented as percent control, with untreated control normalized to 100%.

### Phospho-protein Immunoblotting

For phospho-PKC, phospho-p42/44, and phospho-p38, 10 minute exposure to 20 ng/ml EGF was used as the control. These conditions have been published as optimal for signaling by EGF in several published articles [16,17]. Immunoblotting for phospho-proteins was done by running 30 μg protein, reduced by heating to 80°C for 15 min in the presence of 50 mM DTT, on a 10% Tris-Bis gel (Invitrogen). Protein was transferred to PVDF then blocked with 5% blocker (Biorad) in TBS-T 1 hr. Antibody was diluted 1:1000 in 5% blocker as directed by Cell Signaling, Inc. and rocked overnight, 4°C. Membranes were then washed four times and probed with appropriate HRP-linked antibody (Santa Cruz) 1:5000 for 1 hr. After four TBS-T washes, ECL was done (Amersham). Results were normalized for loading differences by stripping with ImmunoPure IgG elution buffer (Pierce, Rockford IL) for 3 hours at 37°C, then probing for β-actin using an HRP-linked anti-β-actin antibody (Santa Cruz).

### Statistical Analysis

Differences in expression frequency of nAChR subunits at either the RNA or protein level were analyzed by Fisher's Exact Test. In all other experiments, differences from control were determined using Student's T-test. All p-values reflect two-tailed tests.

## Results

### Neuronal and muscle-type nAChR are present on HBE cells and airway fibroblasts

**Figure 1:**
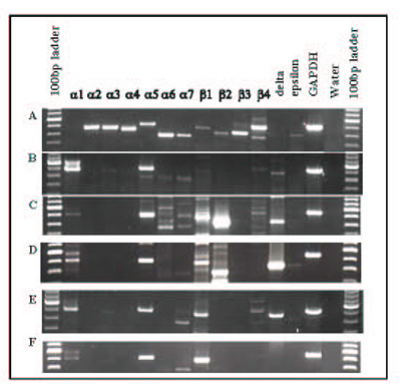
**Expression of nAChR subunits from cell types by RT-PCR. **A) Brain; B) Muscle; C) BEAS2B cell line; D) normal airway fibroblasts; E) human bronchial epithelial cells; F) human bronchial epithelial cells. On each panel, the brightest band on the 100 bp ladder represents 600 bp.

Using RT-PCR, we repeatedly detected mRNA for nAChR subunits in short-term cultures of human airway cells from bronchial biopsies. Figure 1 shows representative PCR products from two HBE cultures (Panel E and F), one airway fibroblast culture (Panel D), and immortalized HBE BEAS2B cells (Panel C). Brain mRNA was used as a positive control for neuronal-type nAChR (Figure 1 Panel A) and muscle mRNA as a positive control for muscle-type nAChR (Figure 1 Panel B), and GAPDH is included on each panel to indicate mRNA quality. A water-only sample was used as a negative control in each experiment. PCR products were found at the expected size for each subunit and were sequenced and compared to the known gene sequences, and confirmed to be the expected sequence for each subunit. HBE cells cultured from airway biopsies from a series of never smokers, ex-smokers, and active smokers were examined by RT-PCR and the results are summarized in Table 3. Due to limited RNA yield, some samples were not examined for all possible subunits. We found that seven different nAChR subunits were expressed in over 50% of the sample examined, including α5, α6, α7, α9, β1, δ and ε. Three additional subunits, α1, α3, and β4 were expressed in at least 30% of cultures examined. The α2 and α4 subunits were also examined, but were never present (data not shown).

**Table 3:** Expression of nAChR in HBE and Bronchial Epithelial Cells Lines as determined by RT-PCR

**HBE**	**α1**	**α3**	**α5**	**α6**	**α7**	**α9**	**β1**	**β2**	**β3**	β4 *	**δ**	**ε**
**Never smoker**	2/9 (22%)	2/8 (25%)	6/9 (67%)	5/9 (56%)	7/9 (78%)	8/8 (100%)	9/9 (100%)	4/7 (57%)	0/8 (0%)	6/9 (67%)	7/8 (88%)	5/9 (56%)
**Active smoker**	3/10 (30%)	0/3 (0%)	9/10 (90%)	3/4 (75%)	7/10 (70%)	3/3 (100%)	9/10 (90%)	5/9 (56%)	0/5 (0%)	1/10 (0%)	6/8 (75%)	7/10 (70%)
**Ex-smoker**	7/18 (39%)	8/19 (42%)	18/19 (95%)	9/19 (47%)	11/18 (61%)	16/18 (89%)	17/19 (89%)	6/16 (38%)	1/19 (5%)	9/19 (47%)	11/18 (61%)	10/19 (53%)
**Total**	12/37 (32%)	10/30 (33%)	33/38 (87%)	17/32 (53%)	25/37 (68%)	27/29 (93%)	35/38 (92%)	15/32 (47%)	1/32 (3%)	16/38 (42%)	24/34 (70%)	22/38 (58%)
**BEAS2B**	+	-	+	+	+	+	+	+	-	+	+	+
**IB3-1**	+	-	+	-	+	+	+	+	-	+	+	+

nAChR subunits present in HBE and bronchial epithelial cell lines as determined by RT-PCR. The table indicates the number of positive cultures out of the total number of cultures examined, as well as the percentage of positive cultures for each subunit. * indicates statistical significance when comparing cultures from never smokers to those of active or ex-smokers.

The subunits that are expressed by HBE cells could potentially combine to form muscle-type (α1/ β1/ δ /ε) heteropentamers, neuronal α7 or α9 homopentamers, and neuronal heteropentamer receptors α3/ α5/ β2 or β4 and α6/β2 or β4. By examining the pattern of mRNA expression in each individual culture, combinations were observed that would produce a functional muscle-type receptor in 7 of 33 (21%) of cultures, a functional α3-containing neuronal type receptor in 10 of 28 (35%) of cultures, α6-containing neuronal type receptor in 13 of 28 (46%), α7 homopentamer receptors in 25 of 37 (68%), and homopentamer α9 receptors in 27 of 29 cultures (93%). Only 2 of 35 (6%) HBE cultures did not express at least one functional nAChR subunit combination.

Two immortalized airway epithelial cell lines also expressed mRNA for many of these nAChR subunits, including muscle-type subunits (Figure 1, Table 3). BEAS2B, derived from normal HBE cells, and IB3-1, derived from the bronchial epithelial cells of a cystic fibrosis patient, were examined. Both immortalized epithelial cultures expressed the subunits required for a functional muscle-type nAChR (α1/β1/δ/ε) and the homopentamers α7 and α9, and BEAS2B cells express mRNA for subunits that may combine to form functional neuronal nAChRs (α6/ β2 or β4).

Airway fibroblasts also expressed nAChR (Table 4). We found that mRNA for α1, α5, α6, α7, α9, β1, β2, δ and ε were all expressed more than 70% of the time. All other subunits were expressed less than one-third of the time. As for HBE cells, we examined the pattern of mRNA expression in each airway fibroblast culture. nAChR subunits could potentially combine to form muscle-type (α1/ β1/ δ / ε) receptors in 77% of fibroblast cultures and both neuronal α7 or α9 homopentamers in 100% of cultures. Neuronal heteropentameric nAChR containing the α6 subunit might be formed in 59% of cultures, and neuronal heteropentamers containing the α3 subunit, with or without the α5 subunit, could combine to form a functional receptor in 27% of cultures.

**Table 4:** Expression of nAChR in Airway Fibroblasts as determined by RT-PCR

**NLFB**	**α1**	**α3**	**α5**	**α6**	**α7**	**α9**	**β1**	**β2**	**β3**	**β4**	**δ**	**ε**
**Never smoker**	5/5 (100%)	2/5 (40%)	5/5 (100%)	5/5 (100%)	5/5 (100%)	5/5 (100%)	5/5 (100%)	5/5 (100%)	2/5 (40%)	2/5 (40%)	5/5 (100%)	5/5 (100%)
**Active smoker**	10/11 (91%)	3/11 (27%)	11/11 (100%)	8/11 (73%)	10/11 (91%)	11/11 (100%)	11/11 (100%)	8/11 (73%)	4/11 (36%)	2/11 (18%)	10/11 (91%)	10/11 (91%)
**Ex-smoker**	9/9 (100%)	0/9 (0%)	8/9 (89%)	5/9 (56%)	5/9 (44%)	8/9 (89%)	6/9 (67%)	6/9 (67%)	2/9 (22%)	3/9 (33%)	9/9 (100%)	8/9 (89%)
**Total**	24/25 (96%)	5/25 (20%)	24/25 (96%)	18/25 (72%)	20/25 (80%)	24/25 (96%)	22/25 (88%)	19/25 (76%)	8/25 (32%)	7/25 (28%)	24/25 (96%)	23/25 (92%)

nAChR subunits present in airway fibroblasts as determined by RT-PCR. The table indicates the number of positive cultures out of the total number of cultures examined, as well as the percentage of positive cultures for each subunit.

The mRNA expression of four nAChR subunits is significantly different when comparing airway fibroblasts and HBE cells. The muscle-type receptor subunits α1, δ, and ε are all expressed more frequently in airway fibroblasts (present in 96%, 96%, and 92%, respectively, of airway fibroblast cultures) than in HBE cells (α1 present in 32% of cultures, δ in 70% of cultures, and ε present in 58% of cultures, p < 0.02 for each subunit). In addition, the β3 subunit is also expressed more frequently in fibroblast cultures than in HBE cells (8/25 compared to 1/28, p < 0.01). The subunit combinations that could form functional receptors were also examined and compared between cell types. Consistent with the individual subunit data, the combination of all subunits for the muscle-type receptor is expressed significantly more frequently in human airway fibroblasts (74%) than in HBE cultures (21%)(p = 0.0001).

The nAChR subunit α9 was frequently expressed by both HBE cells and airway fibroblasts. Although we find this nAChR frequently and it may have physiological significance, it is unlikely that signaling through this receptor would be responsible for the immediate downstream effects seen in our studies, which focus on the effects of nicotine, since nicotine does not act as an agonist for this receptor type.

**Figure 2:**
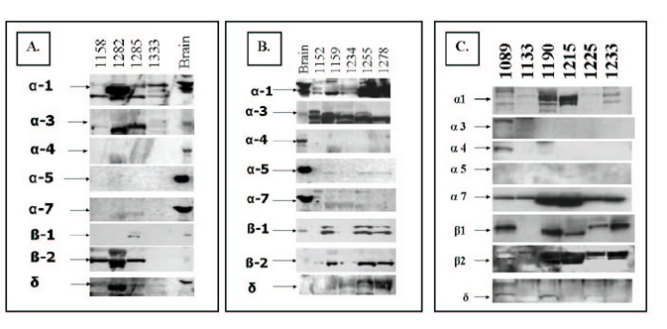
**HBE and airway fibroblasts immunoblotting for nAChR subunits. **Whole brain tissue lysate was used as a positive control. A) nAChR in HBE cells from never smokers; B) nAChR in HBE cells from ex-smokers; C) nAChR in airway fibroblast cells from one ex-smoker (1089), two never smokers (1133,1190), and three active smokers (1215, 1225, 1233). For each subunit, the positive control band was seen at the following size: α1 55 kD, α3 60 kD, α4 70 kD, α5 53 kD, α7 56 kD, β1 59 kD, β2 57 kD, and δ 55 kD.

**Figure 3:**
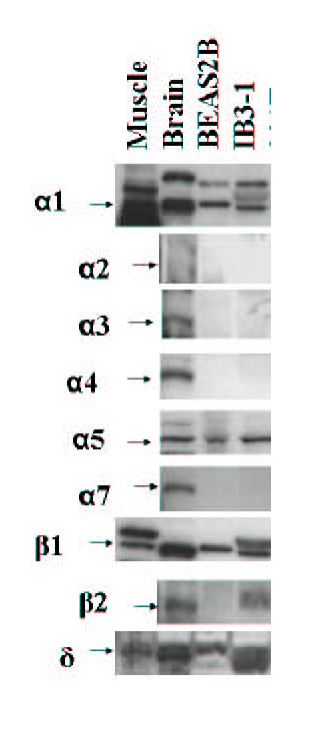
**Expression of nAChR subunit protein by BEAS2B and IB3-1. **Immortalized bronchial epithelial cell lines BEAS2B and IB3-1 were immunoblotted for nAChR. For each subunit, the positive control band was seen at the following size: α1 55 kD, α2 60 kD, α3 60 kD, α4 70 kD, α5 53 kD, α7 56 kD, β1 59 kD, β2 57 kD, and δ55 kD.

We next examined nAChR subunit protein expression using immunoblotting. All the cultures examined expressed protein for nAChR subunits, in general agreement with mRNA expression data (for representative results, see Figure 2, Figure 3). In these experiments, whole brain and muscle tissue lysates as positive controls (Figure 2, Figure 3). For each subunit, the culture was considered positive when a band matching the size of the positive control at the expected kilodalton size was found. A doublet band just above the expected size on the blots strongly suggests that both glycosylated and non-glycosylated forms of the protein are present, and that protein processing is in progress (Figures 2,3). Glycosylation is essential for nAChR folding and expression on the cell membrane, and the higher bands are expected. For the α3 subunit, bands below the expected size were observed which might represent cross-reactivity of the antibody with other nAChR subunits, or protein degradation products, but only those that matched the size of the brain control were considered a positive result. In HBE we found that α1, β2, and δ were usually present (Figure 2A,2B). Although there are also lower cross-reacting bands for these nAChR subunits, a band of the correct size was also frequently seen, and upper bands suggest that the glycosylated form of the subunits are present (Figure 2A,2B). Protein for the subunits α4 and α7 were not observed in HBE, and α3, α5, and β1 were sometimes present, although α5 was expressed at a barely detectable level compared to other subunits (Figure 2B).

To examine these findings more generally, nAChR protein was examined in additional HBE cultures from never smokers, active smokers, and ex-smokers (Table 5). Only some of the cultures examined by immunoblot were derived from the same individual as those we had examined by RT-PCR, so a direct comparison with the mRNA expression results was not usually possible. Subunit protein expression is generally consistent with our previous results, showing that the muscle-type receptor subunits α1, β1, and δ are detected at the protein level and theoretically form a functional receptor together with ε. Although we have not found an antibody for ε that will detect this protein in our positive controls, RT-PCR results indicate that it is frequently expressed (Table 3).

**Table 5:** Expression of nAChR in HBE and Bronchial Epithelial Cell Lines as determined by Immunoblot

**HBE**	**α1**	**α3**	α5*	**α7**	**β1**	**β2**	**δ**
**Never smoker**	5/8 (63%)	1/4 (25%)	0/4 (0%)	0/4 (0%)	1/4 (25%)	3/5 (60%)	3/4 (75%)
**Active**	2/2 (100)	n.d.	2^+^/2 (100%)	0/2 (0%)	2/2 (100%)	2/2 (100%)	n.d.
**Ex-smoker**	5/7 (71%)	1/5 (20%)	5^+^/7 (71%)	0/7 (0%)	4/7 (57%)	5/5 (100%)	5/5 (100%)
**Total**	12/17 (71%)	2/9 (22%)	7/13 (54%)	0/13 (0%)	7/13 (54%)	10/12 (83%)	6/6 (100%)
**BEAS2B**	+	-	+	-	+	-	+
**IB3-1**	+	-	+	-	+	+	+

nAChR subunit protein present in HBE and bronchial epithelial cell lines as determined by immunoblot. The table indicates the number of positive cultures out of the total number of cultures examined, as well as the percentage of positive cultures for each subunit. * indicates statistical significance when comparing never smoking samples to combined active and ex-smoker samples. ^+ ^The α5 subunit was present in these samples, but at low levels compared to other subunits and control.

One difference between our RT-PCR results and our immunoblotting is in the frequency of α7 expression. α7 mRNA was expressed in 68% of our HBE cultures, but the protein for this subunit was never detected, although the protein was detected in positive controls and in airway fibroblasts. This subunit is either transcribed but not translated, or the protein may be expressed below the limit of detection for immunoblotting in HBE cells.

We analyzed our protein data for combinations of subunits that would form functional receptors. We found that all three of the subunits needed for a functional muscle receptor were highly expressed at the protein level in 44% of the HBE cultures examined. The other functional combinations analyzed at the protein level are the neuronal α3-containing heteropentamers that were present in 33% of the cultures.

We also determined that nAChR subunit protein is expressed in airway fibroblasts (Figure 2). We found that subunit expression had less variation among cultures from different individuals in fibroblasts than HBE cultures. The α1, α7, β2, and δ subunits were expressed in 100% of cultures. The α3, α4, and α5 were never expressed, and β1 was expressed in 83% of cultures. The frequency of expression of α7 was significantly higher in fibroblasts than HBE cultures (100% versus 0%, p < 0.0001), and α5 is more frequently expressed in HBE cultures (54% versus 0%, p < 0.05).

We also found that nAChR subunit protein is expressed on two cell lines derived from normal bronchial epithelial cells described above (IB3-1, BEAS2B). Figure 3 is a representative immunoblot and Table 5 contains the complete table of results that have been repeated in independent experiments. We find that both cell lines express α1, β1, and δ protein. Therefore, the muscle-type (α1/ β1/ δ / ε) is most likely the major functional nAChR on BEAS2B and IB3-1. Neither cell line expresses protein for the α7 homopentamer. BEAS2B cells also express protein for α5 and β2 but do not appear to have another appropriate heterodimer partner, such as α3, to produce a functional neuronal nAChR. Based on the protein results, the only pentameric nAChR receptor detected in the BEAS2B and IB3-1 epithelial cell lines is the muscle-type heteropentamer.

### NAChR are functional in lung epithelial cells

**Figure 4:**
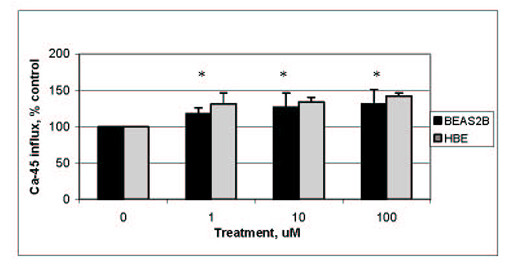
**Calcium influx after exposure of HBE or BEAS2B cells to nicotine. **Intracellular calcium-45 was measured after exposure to nicotine for 5 minutes. * statistically significant as compared to untreated control.

We examined functionality of the AChR on HBE cells by measuring calcium influx. After treatment with nicotine, extracellular radioactive calcium (Ca-45) is internalized by BEAS2B cells and short-term HBE cultures (Figure 4). BEAS2B cells were chosen for these experiments instead of IB3-1 due to the derivation of the cells. Since IB3-1 cells were derived from a cystic fibrosis patient with the classic sodium-channel defect, and nAChR can also act as sodium channels, their expression or function may be altered in this cell type due to abnormal ion levels. A derivative of the calcium ionophore myomycin was used as a positive control. Calcium-45 levels were 5 times over control levels using the positive control (data not shown). This high level of calcium influx is associated with loss of membrane integrity caused by ionophore.

The calcium influx seen in our experiments occurs within 5 minutes of treatment with nicotine and at concentrations from 1 μM to 100 μM in both cell types. Calcium influx was dose-dependent and reached 131% of control in BEAS2B cells and 137% of control in HBE cells. At all doses, influx of calcium-45 was statistically greater than control in BEAS2B cultures (p < 0.05).

**Figure 5:**
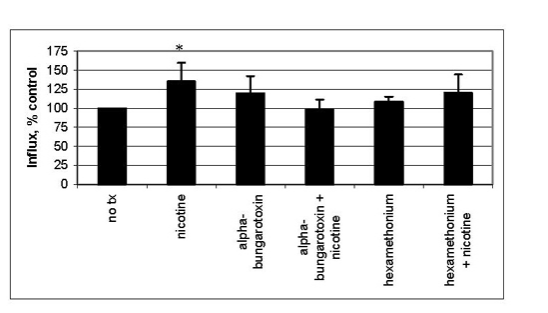
**Calcium influx after exposure of BEAS2B cells in the presence of antagonist. **BEAS2B cell were exposed to 1 μM nicotine in the presence or absence of nAChR antagonist. Intracellular calcium-45 was measured after exposure to nicotine for 5 minutes. * statistically significant as compared to untreated control.

To test the specificity of this response, we used the nicotinic antagonists α-bungarotoxin and hexamethonium with BEAS2B cells and nicotine. α-Bungarotoxin will block muscle-type nAChR as well as α7 homopentamers and hexamethonium will block neuronal heteropentamer nAChR such as α3- and α6- containing receptors. We used ionophore alone and with the nicotinic antagonists as a control in these experiments. Antagonists had no effect on ionophore-induced calcium influx (data not shown). In this experiment we found that α-bungarotoxin could completely prevent the calcium-45 influx seen after nicotine treatment, while hexamethonium could only slightly inhibit the effect of nicotine in BEAS2B cells (Figure 5). Based on this data, the neuronal nAChR α3- and α6- containing receptors do not appear to play a significant role in controlling calcium influx after nicotine treatment since the inhibitor of this receptor type, hexamethonium, could not significantly inhibit influx. Consistent with our immunoblot data, the influx of calcium after exposure of BEAS2B cells to nicotine is likely mediated through muscle-type receptors (α1/β1/δ/ε), and can be blocked by the inhibitor specific to these receptor types, α-bungarotoxin.

### Signaling pathways are activated in response to nicotine

We determined if protein kinase C (PKC) responds to nicotine because it is commonly activated by calcium influx. Calcium influx is an immediate effect of nicotine exposure to BEAS2B cells (Figures 4 and 5). The mitogen-activated protein kinase (MAPK) family members p38 and p42/44 were also examined because previous data showed that nAChR may be involved in regulation of apoptosis and growth [1,9]. In examination of PKC and MAPK, cells were treated with epidermal growth factor (EGF) as a positive control. We chose actin to correct for total protein for densitometry so that we could probe blots for a number of signaling pathways without compromising the protein on blots with unnecessary stripping procedures.

**Figure 6:**
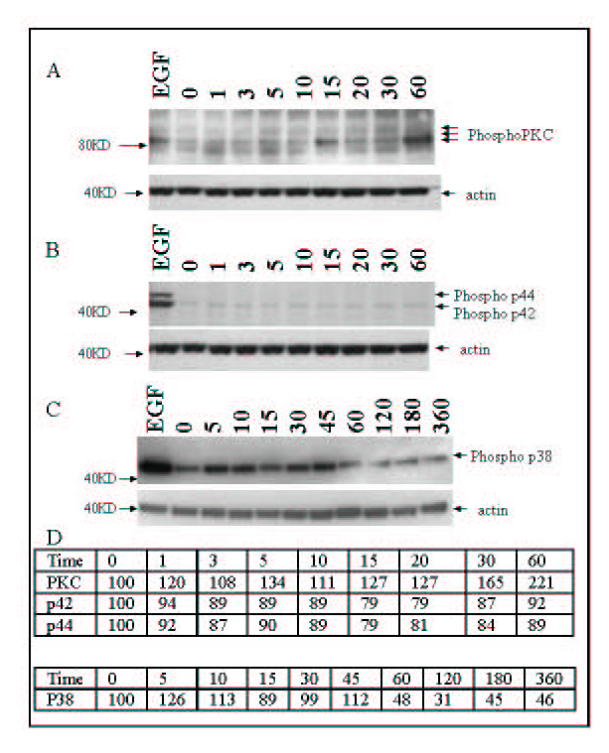
**Phosphorylation of proteins after nicotine exposure in BEAS2B cells. **Nicotine causes phosphorylation of PKC and p38, but not p42/44 in BEAS2B cells. Cells were exposed to 100 μM nicotine for the times indicated. Panel A) represents the effect of nicotine on phosphorylation of PKC; B) represents the effect of nicotine on phosphorylation of p42/44; C) represents the effect of nicotine on phosphorylation of p38 D) is densitometry for the immunoblots expressed as percent of untreated control after correction with actin.

Using phosphorylation as a marker of activation, we find that PKC and p38, but not p42/44 are activated after treatment with nicotine in BEAS2B (Figure 6). As shown in the time course in Figure 6, phosphorylation of PKC is above control levels at 15 minutes of continuous treatment and stays above control through the longest time point, 60 minutes of continuous treatment. After correction for total protein using actin, densitometry shows that PKC levels are 120% of control after 1 minutes of exposure, and after 60 minutes are 221% of untreated control. In this experiment, a pan-phospho-PKC antibody was used that detects six isoforms of phosphorylated PKC between 78 and 85 KD. P38 was also phosphorylated immediately after nicotine treatment, with band intensity of 126% of control at the first timepoint examined, 5 minutes. Unlike PKC, phosphorylation of p38 was only briefly present, and phosphorylation drops to below control by 15 minutes of treatment. When probed for phospho-p42/44, phosphorylation state was never above control in BEAS 2B through 60 minutes of treatment (Figure 6).

**Figure 7:**
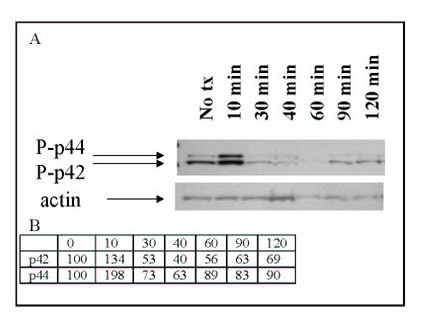
**Nicotine causes phosphorylation of p42/44 in airway fibroblasts. **Cells were exposed to 100 μM nicotine for the times indicated. Panel A) represents the effect of nicotine on phosphorylation of p42/44; B) is densitometry for the immunoblots expressed as percent of untreated control after correction with actin.

In contrast, signaling experiments done with short-term airway fibroblast cultures show that nicotine caused phosphorylation of p42/44 within 10 minutes of exposure (Figure 7). After densitometry and correction for loading differences, phosphorylation was increased to 198% of control at the 10 minute timepoint. Similarly to phosphorylation of p38 in BEAS2B cells, phosphorylation is tightly controlled, and returns to below control levels by 30 minutes. Use of α-bungarotoxin showed that phosphorylation of p42/44 can be blocked by this nAChR antagonist (data not shown), indicating that muscle-type and/or α7 receptors are involved in this response.

Together, these data indicate that the muscle-type (α1/β1/δ/ε) nAChR is consistently present on airway epithelial cells, while airway fibroblasts consistently demonstrate both a muscle-type and an α7 homomeric nAChR. Normal airway epithelial cells may also sometimes express the neuronal α3/α5/β2 or α6/β2 nAChR. The nicotinic receptors are functional, regulate calcium influx upon ligand binding, and lead to downstream activation of the signaling pathways MAPK or PKC when bound by nicotine. Downstream effects can be blocked by use of nicotinic antagonists.

### Long-term nicotine exposure and nAChR expression

We examined the relationship of prior smoking to nAChR expression on airway cells. To do so, we compared subunits expressed at the mRNA and protein level in HBE cultures from never smokers, active smokers, and ex-smokers to determine if long-term exposure to nicotine was a factor in the type of nAChR expressed. As shown in Table 3, comparison of HBE cultures from 9 never smokers and 10 active smokers indicates that active smoking was associated with a significant decrease in mRNA expression of the β4 subunit (6 of 9 never smokers expressed this subunit compared to 1 of 10 active smokers (p = 0.02). This difference was not significant when comparing active to ex-smokers. In addition, frequency of expression in HBE cells of other subunits that make up the neuronal nAChR was not significantly different between active and never smokers. At the protein level, our data show that α5 may be upregulated in HBE cells at the protein level in response to chronic nicotine exposure (Table 5). Fewer never-smokers express α5 protein than ever-smokers (p < 0.05), although at lower levels than its receptor partners. An available antibody to the β4 subunit was not found to be specific to that subunit in our controls, therefore we could not determine if the β4 protein is modulated by tobacco exposure, as observed at the mRNA level.

Interestingly, in airway fibroblasts mRNA patterns for combinations of neuronal heteropentamers containing the α3 subunit were downregulated with smoking (Table 4). Cultures containing mRNA for all the subunits to form α3/ β2 or β4 receptors, with or without α5, were observed in 80% of never-smokers, but only 25% of active smokers and none of the ex-smokers examined. This difference was significant when comparing never smokers to ex-smokers or comparing never-smokers to ex- and active smokers together (p < 0.01). However, when analyzing protein results, no differences were found among active-, ex-, and non-smokers in the projected receptor type.

## Discussion

Recent evidence suggest that endogenous acetylcholine is a local signaling molecule in non-neuronal tissue, and that nicotinic acetylcholine receptors are found outside the nervous system. Our data show that HBE cells can express mRNA for the neuronal α3/α5/β2 or β4, α6/β2 or β4, and α7 and α9 pentamers, as well as the muscle type α1/β1/δ/ε nAChR. Previous data in the human airway examined small numbers of HBE cultures for a limited number of nAChR subunits. Maus *et al. *[12] used RT-PCR and binding studies to show that the α3/α5/β2 nAChR were present on HBE cells. West *et al. *[9] also characterized nAChR on three non-immortalized bronchial epithelial cell lines and found the α3/α5/β2 nAChR subunits as well as α7, α9, and α10. However, neither investigation examined HBE cultures for the presence of the muscle-type nAChR. West *et al. *[9] examined α1 mRNA expression by RT-PCR, which was negative, consistent with our data that indicate that the mRNA for the α1 subunit is expressed in approximately one-third of HBE cultures from different individuals. In the present study, we have determined that several types of nAChR are consistently present on many primary cultures of HBE cells. Using a panel of 38 HBE cultures for RT-PCR analysis, we find that although not every nAChR subunit RNA is present in every culture, the mRNA for seven different nAChR subunits are consistently expressed in combinations that could combine to form both muscle and neuronal-type receptors. Immunoblotting confirmed that the nAChR mRNA for these subunits was translated into detectable protein. Not all subunits that are expressed at the mRNA level are highly expressed at the protein level, indicating that some regulation of nAChR expression may occur at the translational and post-translational levels. This has been previously shown in neuronal cells, where transcripts for the α7 subunit are present even on cells without functional α7 receptors [18]. Our data indicate that this also occurs in HBE cells, where α7 transcripts are frequently found, but protein of the correct size is not. Thus the conclusion in prior literature that various neuronal nAChR receptors, including α7, are responsible for actions of nicotine in HBE may not be definitive.

Instead, our results suggest that the muscle-type nAChR present in HBE cells may have a functional role in HBE cells that has not previously been considered. The muscle-type receptor was more recently characterized than the neuronal type and previous literature never examined HBE cultures for the muscle-type receptor [9,10,12]. This finding was further supported by the observation that two immortalized cell lines derived from HBE also expressed protein for the muscle-type receptors, while lacking the α7 homopentamer protein.

Similarly to HBE, mRNA and protein for nAChR subunits are commonly expressed by airway fibroblast cultures. Airway fibroblasts have never been examined for the presence of nicotinic receptors. Dermal fibroblasts have been shown to express mRNA for some receptors, although they were not examined for muscle-type receptors [19], and gingival fibroblasts respond to nicotine by decreasing expression of integrins and increasing expression of c-fos, but they have not been characterized for nAChR [20-22]. We find that airway fibroblasts express all the subunits required for muscle-type nAChR. In contrast to HBE, these cells also express readily detectable protein for the α7 receptor in all the cultures examined. Therefore, in these cells, the muscle-type receptor and the α7 neuronal homopentamer are the major functional nAChR that may be responsible for signaling initiated by nicotine. α7 receptors are less susceptible to inactivation in the long-term presence of agonist, while muscle-type receptors are more likely to undergo inactivation [23]. Therefore, in cells with these different receptors, there may be differences in the initiation of downstream signaling pathways especially in response to extended exposure to nicotine, such as occurs in a chronic smoker.

Calcium influx is a hallmark of the opening of the nAChR ion channel. An increase in intracellular calcium from the extracellular milieu can occur either by a direct influx of calcium through the nAChR channel, as occurs in α7 receptors, or by an influx of sodium that leads to depolarization of the cell and the opening of L-channels, as occurs after agonist binding to heteropentamer nAChR [24,25]. We determined that nAChR are functional in both BEAS2B and HBE cells; treatment of cells with nicotine leads to an influx of extracellular calcium. The influx of calcium seen after nicotine treatment was similar in magnitude to calcium influx seen in neuronal cells after activation of L-channels (144%) [26]. Additionally, we differentiated between functional muscle-type receptors and neuronal heteropentamer receptors. In BEAS2B cells, calcium influx stimulated by nicotine was significantly inhibited by a muscle/ α7 antagonist, but only slightly inhibited by a neuronal heteropentamer antagonist. This confirms the observation that the neuronal heteropentamer nAChRs do not play a major functional role in the response of BEAS2B cells to nicotine in our hands. Since α7 protein was not detected in HBE or BEAS2B cells, the muscle-type nAChR is more likely the major functional receptor type.

Nicotine has previously been shown to affect signaling in human airway cells, and acetylcholine, the endogenous ligand, causes proliferation of HBE cells [1,9]. In our experiments, nicotine initiated signaling pathways involved in cell growth and apoptosis in both HBE cells and airway fibroblasts. In BEAS2B cells, immediate phosphorylation of PKC is likely due to calcium influx. Calcium is a known cofactor for classical PKC activation and is required along with binding to diacylglyerol (DAG) for functional conformation. Phosphorylation of PKC, consistent with activation, increases over time. This may indicate that upregulation of other PKC cofactors, such as DAG, may occur as downstream events after calcium influx, leading to an enhanced PKC signal over time.

Over the same time period, the MAPK family member p38 but not p42/44 is phosphorylated, a required step for activation. The activation of p38 is associated with regulation of apoptosis in response to cellular stress. The MAPK family kinases, such as p38, are not known to be directly activated by calcium; however there are several indirect pathways that lead to rapid phosphorylation of these pathways. These include signaling through the calcium/calmodulin-dependent protein kinases CaMKI, CaMKII, and CaMKIV as well as the calcium-activated signaling molecule PYK2.

This finding is in contrast to signaling activated by nicotine in airway fibroblasts. These cells phosphorylate p42/44 immediately upon treatment with nicotine. Like p38, p42/44 is not directly activated by calcium, but could be phosphorylated by calcium-activated signaling molecules. However, the pathways that lead to phosphorylation of different MAPK family members in the two cell types in response to nicotine have not been elucidated. It is possible that the nAChR type differences are responsible for the differential MAPK effects. For example, the α7 receptor is highly expressed on airway fibroblasts but not on HBE cells. The additional nAChR type on these fibroblasts may change the signaling pathways activated by cells in response to nicotine. This is consistent with a previous study by Jull *et al *[27]. This study showed that ligand binding to the α7 nAChR in SCLC cells leads to activation of p42/44 through Raf [27]. A similar pathway may be activated by the binding of nicotine to the α7 nAChR in airway fibroblasts.

Finally, we found that smoking had only modest effects on nAChR expression in the airway. Previous studies in the brain indicate that certain nAChR may be increased in frequency in smokers as compared to non-smokers [28], and a published experiment showed that there was an increase in the α3 nAChR expressed in respiratory epithelial cells in one smoker compared to one nonsmoker [10]. Due to the limited sample size in the study of lung cells, it is impossible to know if this difference was due to individual variation or was a smoking-induced change, especially when considering the variability of nAChR subunit expression seen among individuals in our study. In this study of HBE cultures using a panel of active smokers, never smokers, and ex-smokers, there were some statistical differences in the mRNA and protein expression of nAChR subunits in cultures derived from donors with different smoking histories, including the increased presence of protein for an α5 subunit in smokers that could combine with the α3 containing receptors and change the calcium permeability [29]. However, the α5 subunit was expressed at a much lower protein level than the α3/β2 subunits, and may only be present in some of the neuronal-type receptors. Other changes that were documented with smoking status did not occur in the major functional nAChR of the cell type. Corresponding changes also did not occur in the other subunit partners that are necessary for function, so the effect on overall functional receptor expression was probably unchanged with smoking status in HBE cells.

In contrast, there was a significant decrease in the frequency of the expression of the functional combination of subunits for the α3-containing receptors in airway fibroblasts of smokers and ex-smokers compared to never-smokers. This nAChR type is a sodium channel that undergoes inactivation upon long-term exposure to agonist, and, in airway fibroblasts, appears to be downregulated with long-term exposure to nicotine. This change in receptor expression remains even after exposure to nicotine ceases, as evidenced by the reduced frequency of expression in ex-smokers.

## Conclusions

We have shown that short-term cultures of normal airway fibroblasts as well as normal human bronchial epithelial cells from a number of different human donors consistently express functional nAChR and that these cell types differ in the type of nAChR they express. It is likely that the muscle-type nAChR plays a major role in the response of HBE cells to nicotine, and that the neuronal heteropentamers play a more minor role. Calcium influx as well as initiation of downstream signaling pathways indicate that receptors are functional and that both human bronchial epithelial cells and airway fibroblasts respond to nicotine, and those signaling responses may differ due to the difference nAChR present on the cell type. Together, these data suggest that exposure of the human airway to nicotine through tobacco smoke may have physiological consequences on airway homeostasis involving both the airway mucosa and the underlying submucosal mesenchymal cells. As such, nicotine may act to promote lung disease by acting to change cell growth and apoptosis. In airway fibroblasts this may leading to thickening of the airway wall seen in the pathogenesis of COPD. In the bronchial epithelium this may lead to preneoplasia or development of frank cancer.

## Abbreviations

nAChR: nicotinic acetylcholine receptor

HBE: human bronchial epithelial cell

PKC: protein kinase C

MAPK: mitogen activated protein kinase

BEGM: bronchial epithelial growth medium

EGF: epidermal growth factor

P42/44: Extra-cellular signal regulated kinase isoforms 1 and 2

## Authors' Contributions

DLC designed and performed the majority of the experiments and data analysis, and wrote the manuscript. TMH designed and performed RT-PCR with the assistance of MJS. JDL and NAC contributed the tissues that were grown into primary cultures and provided information on smoking history. AGD cultured the primary cells. JMS conceived of the study and participated in its design and coordination. All authors read and approved the manuscript.
